# Efficacy of Selinexor in Relapsed/Refractory Multiple Myeloma (RRMM) Patients with del17p and Other High-Risk Abnormalities (A Retrospective Single-Center Study)

**DOI:** 10.3390/life14030384

**Published:** 2024-03-14

**Authors:** Hamid Ehsan, Myra Robinson, Peter M. Voorhees, Kristen Cassetta, Shanice Borden, Shebli Atrash, Manisha Bhutani, Cindy Varga, Mauricio Pineda-Roman, Reed Friend, Barry A. Paul

**Affiliations:** 1 Levine Cancer Institute, Atrium Health Wake Forest Baptist, 4525 Cameron Valley Pkwy Suite 3500, Charlotte, NC 28211, USA; hamid.ehsan@atriumhealth.org; 2 Department of Biostatistics and Data Sciences, Levine Cancer Institute, Atrium Health Wake Forest University School of Medicine, Charlotte, NC 28204, USA; 3 Department of Hematologic Oncology and Blood Disorders, Levine Cancer Institute, Atrium Health Wake Forest University School of Medicine, Charlotte, NC 28204, USA

**Keywords:** Selinexor, RRMM, high-risk multiple myeloma

## Abstract

Selinexor (Seli) is a first-in-class, oral selective inhibitor of the nuclear export protein, exportin-1 (XPO1). Seli exhibits its antitumor effect through the blockage of XPO1, which increases nuclear retention of tumor suppressor proteins (TSPs), including p53, thereby limiting the translation of oncogenes, triggering cell cycle arrest and the death of malignant cells. Multiple Myeloma (MM) patients with del17p are deficient in TP53 and have a particularly poor prognosis. Given its unique mechanism of action, we investigated whether Seli has increased efficacy in RRMM patients with del17p compared to other high-risk cytogenetics (OHRC). This is an IRB-approved observational study of RRMM patients with high-risk cytogenetics (del17p, t (4;14), t (14;16) or gain 1q) or standard-risk cytogenetics treated at the Levine Cancer Institute (LCI) with a Seli-based regimen between January 2019 and December 2022. Time-to-event endpoints (PFS, OS) were evaluated using Kaplan–Meier (KM) methods. Log-rank tests compared time-to-event endpoints between cohorts [del17p vs. OHRC vs. standard risk]. We identified 40 RRMM patients with high-risk cytogenetics, including 16 patients with del17p and 24 patients with OHRC, as well as 20 with standard-risk cytogenetics. The median age was 62.5 vs. 69 vs. 65.5 years (del17p group vs. OHRC vs. standard risk). The median prior line of therapies was five (range: 3–16) with similar rates of prior autologous stem cell transplant in all arms (68.8% vs. 62.5% vs. 70.0%). The most frequently used regimens were Seli–Pomalidomide–dexamethasone(dex) or Seli–Carfilzomib–dex (Seli-Kd) in the del17p group and Seli-Kd in the OHRC and standard-risk groups. The median time to start the Seli-based regimen after initial MM diagnosis was 5.6 years for the del17p group, 4.1 years in OHRC, and 4.8 years in the standard-risk group. The median follow-up time after the start of the Seli-based regimen was 10.5 months (mos) in the del17p group, 8.4 mos in OHRC, and 10.3 mos in the standard-risk group. In the del17p group, 50% had an objective response, 41.7% in the OHRC, and 35% in the standard-risk group (*p* = 0.71). Depth of response was also similar across the arms (12.5% vs. 12.5% vs. 10.0% VGPR *p* = 0.99). The median OS was 10.9 mos in the del17p group, 10.3 mos in the OHRC, and 10.3 mos in the standard-risk group (*p* = 0.92). The median OS was 15.5 mos for patients who received Seli as a bridging therapy versus 9 mos for Seli use for other reasons rather than as a bridge. Overall, Seli-based regimens showed promising responses even in this heavily pretreated population. Our analysis suggests that Seli-based regimens lead to similar outcomes among RRMM patients with del17p, OHRC, and standard-risk cytogenetics. This contrasts with previously reported outcomes using combinations of novel therapies in this population, where the del17p patients often have a poorer prognosis. Interestingly, our data suggest that Seli is a particularly effective bridging modality for patients preparing for CAR-T cell therapies in our population. Further investigation into this population is warranted, including in earlier lines of therapy, in hopes of seeing a more durable response.

## 1. Introduction

Selinexor (KPT-300) is a first-in-class, oral selective inhibitor of the nuclear export protein, also known as exportin-1 (XPO1) or chromosomal maintenance-1 (CRM1) [1]. XPO1 attaches to the guanosine triphosphate (GTP)-binding nuclear protein known as Ran and forms a complex of XPO1/Ran GTP nucleocytoplasmic transport protein which is responsible for the extra nuclear transport of many tumor-suppressor proteins (TSPs), such as p53 and breast cancer genes 1 and 2 (BRCA1/2) [2,3]. The overexpression of XPO1 can lead to the aberrant distribution of these regulatory proteins with increased localization to the cytoplasm, resulting in an increased translation of oncoprotein mRNAs and functional inactivation of TSPs. This selects for the evasion of apoptosis and relatively unchecked proliferation in malignant cells leading to a more aggressive and refractory clinical phenotype [4]. XPO1, is overexpressed in multiple myeloma (MM) cells and is essential for myeloma cell survival [5]. The overexpression of XPO1 leads to the aberrant distribution of common regulatory proteins—especially p53 [6,7]. By inhibiting XPO1, Selinexor causes the nuclear retention of tumor-suppressor proteins, thereby limiting translation of oncogenes and leading to cell cycle arrest and cell death [5,8].

The gene for human p53 cellular tumor antigen (TP53) is located on the short arm of chromosome 17 (17p13). In normal physiological circumstances, the p53 signaling pathway is largely dormant, with low levels of the p53 protein produced. In times of increased cellular stress such as hypoxia, DNA damage, or cellular injury, the p53 pathway becomes activated, resulting in an increased accumulation of the nuclear p53 protein and leading to cell cycle arrest, apoptosis, and inhibition of angiogenesis in an effort to preserve genomic integrity [9,10,11].

The loss of TP53 function either by the deletion of the short arm of chromosome 17 (del17p) or through inactivating mutations is a relatively common event in multiple myeloma (MM) and increases in frequency with disease progression [12,13,14,15]. Loss of TP53 function portends a worse prognosis and increased risk for treatment resistance and represents a high-risk population that is often underrepresented in clinical trials, limiting our ability to elucidate the optimum treatment regimen [15,16,17,18].

There is growing evidence that XPO1 plays a role in drug resistance involving many malignancies. There are several molecular XPO1 cargoes that might contribute to drug resistance by affecting tumor suppressor (TP53, BRCA1) and survival proteins (MCL1) [19]. Selinexor can help in improving the drug sensitivity of resistant cells by resorting the function of these proteins and by facilitating the expression of DNA-damage repair proteins [20]. Selinexor can lead to the nuclear accumulation of p53, which leads to accelerating myeloma cell death [21].

Selinexor is currently FDA approved in combination with dexamethasone for the treatment of relapsed refractory multiple myeloma (RRMM) in adult patients who are treated with at least four prior therapies, and in combination with bortezomib and dexamethasone for patients who have received at least one prior line of treatment [22,23]. Given the unique mechanism of action of Selinexor, we investigated whether it may have increased efficacy in RRMM patients with del17p compared to MM patients with other high-risk cytogenetics (OHRC). 

## 2. Methods

This is an observational study of RRMM patients with high-risk cytogenetics [del17p, t(4;14), t(14;16) or gain/amplification of 1q] and standard-risk cytogenetics treated at Levine Cancer Institute (LCI) with a Selinexor-based regimen between January 2019 and December 2022. Our protocol and all amendments were approved by the institutional review board. We included patients in our analysis if they had received at least one full 28-day cycle of a Selinexor-based regimen with 6 months of follow up from the start of treatment or experienced a PFS event within 6 months of starting a Selinexor-based therapy. The outcomes of interest included an objective response (defined as partial response or better), progression-free survival (defined as time from Selinexor start to progression or death or censored at last disease assessment), and overall survival (defined as time from Selinexor start to death or censored at last contact). Numerical data were summarized with descriptive statistics, including medians and ranges, while categorical variables were summarized with frequencies and proportions. Time-to-event endpoints, such as progression-free survival (PFS) and overall survival (OS) were evaluated using Kaplan–Meier (KM) methods with median survival and landmark rates estimated from KM curves. Log-rank tests compared time-to-event endpoints between cohorts [del17p versus other (non-del17p) high-risk (OHRC) versus standard-risk cytogenetics]. Fisher’s exact tests compared response endpoints between cohorts.

## 3. Results

We identified 60 patients with RRMM who were treated with a Selinexor-based regimen and have cytogenetic information available. Twenty patients had standard-risk cytogenetics and forty patients had high-risk cytogenetics. The population with high-risk cytogenetics included 16 patients with del17p and 24 patients with other high-risk cytogenetics (OHRC). Patients with del17p in addition to another high-risk cytogenetic feature were included in the del17p group. The baseline characteristics of the included patients are summarized in Table 1. The median age was similar across cohorts (65.5 vs. 62.5 vs. 69 years, standard risk versus del17p group versus OHRC, respectively). The proportion of males was consistent across cohorts. The OHRC cohort had a higher proportion of Black patients (41.7%) than the other cohorts (18.8% in del17p cohort, 20.0% in standard risk cohort). ISS and R-ISS were similar between the del17p and OHRC cohorts (ISS III: 46.2% versus 47.8%; R-ISS III: 38.5% versus 35.0%, respectively). In the standard-risk cohort, 14.3% were ISS III and 0% were R-ISS III. The most commonly used dose of Selinexor was 80 mg weekly, followed by 60 mg weekly, and 100 mg weekly.

Selinexor-based regimens were used as a bridging therapy in 12.5% of patients with del17p, 25% with OHRC, and 10% with standard-risk cytogenetics. Across all cohorts, the most frequently used regimen was Selinexor–carfilzomib(K)–dexamethasone (Table 2). All patients were heavily pretreated and refractory to numerous therapies. The median number of prior lines of therapy was four (range 3–11) for del17p patients, five (range: 3–12) for patients with OHRC, and five (range 3–16) for patients with standard risk cytogenetics. Most patients had undergone a prior autologous stem cell transplant (68.8% with del17p, 62.5% with OHRC, 70% with standard-risk cytogenetics). In the del17p group, 93.3% patients were refractory to carfilzomib and all patients (100%) were refractory to lenalidomide, bortezomib, pomalidomide, and an anti-CD38 antibody. For the OHRC group, 81.8% of patients were refractory to lenalidomide, 87.5% to bortezomib, 90.5% to carfilzomib, 95.2% to pomalidomide, and 95.8% to anti-CD38 antibody. Finally, rates of refractoriness in the standard-risk group were 95% to lenalidomide, 75% to bortezomib, 88.9% to carfilzomib, 100% to pomalidomide, and 94.7% to an anti-CD38 antibody.

The median time since MM diagnosis was 5.6 years (range: 0.7–25.2) in del17p patients, 4.1 years (0.8–11.5) in OHRC patients, and 4.8 years (1.0–11.2) in standard-risk cytogenetics patients. The median follow up was 10.5 months (range: 2.5–33.0) in del17p patients, 8.4 months (0.6–49.1) in OHRC patients, and 10.3 months (0.7–54.5) in patients with standard-risk cytogenetics.

**Figure 1:**
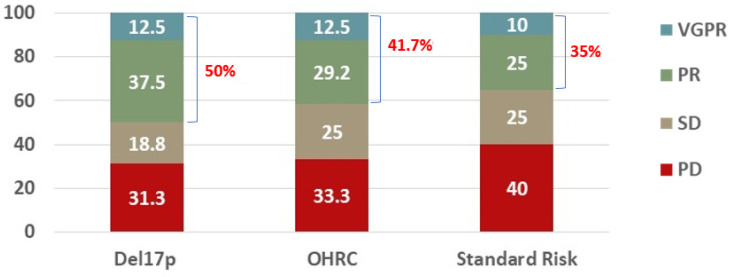
Best response to a Selinexor-containing regimen in RRMM patients with del17p (N = 16), other high-risk cytogenetics (N = 24), or standard risk (N = 20). Overall response rate (ORR) defined as patients who achieved at least a partial response (PR) is listed in red.

The objective response rate was similar between the cohorts (50% with del17p, 41.7% with OHRC, 35% with standard-risk cytogenetics, *p* = 0.714). The distributions of responses were similar between the cohorts (Figure 1, *p* = 0.990). There was no statistically significant difference in the rates of a very good partial response (VGPR) (12.5% vs. 12.5% vs. 10%), a partial response (PR) (37.5% vs. 29.2% vs. 25%), stable disease (SD) (18.8% vs. 25% vs. 25%), or progressive disease (PD) (31.3% vs. 33.3% vs. 40%) noted between patients with del17p, OHRC, and standard-risk cytogenetics, respectively (*p* = 0.990).

**Figure 2:**
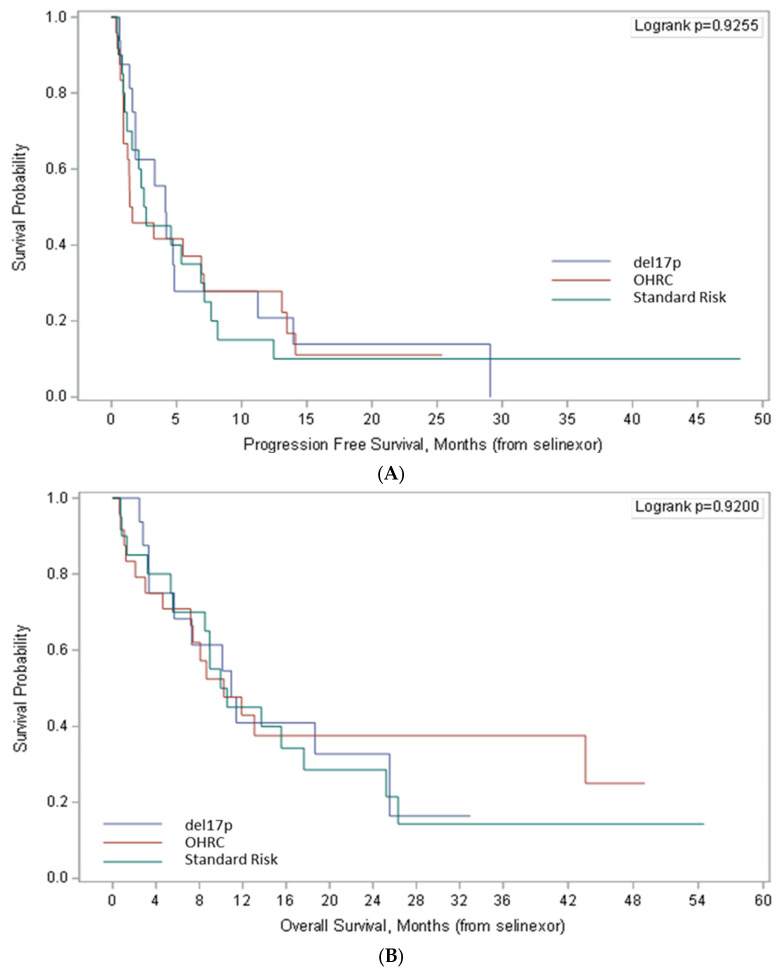
Progression-free survival (**A**) and overall survival (**B**) in patients del17p, other high-risk cytogenetics (OHRC), and standard-risk cytogenetics treated with Selinexor-containing regimens. Survival outcomes were similar between cohorts, contrary to most agents which have lower efficacy in del17p patients.

The median progression-free survival (PFS; Figure 2A) was numerically higher (4.1 months; 95% Cl, 1.6–11.2) in the del17p group as compared to the OHRC group (1.5 months; 95% Cl, 0.9–7.10) and the standard-risk cytogenetics group (2.6 months; 95% Cl, 1–17.1), but not significantly different (*p* = 0.926). The 6-month PFS was 27.8% (95% CI: 8.8–51%) for the del17p group, 37% (95% CI: 18.5–55.7%) for the OHRC group and 35% (95% CI: 15.7–55.2%) for the standard-risk cytogenetics group. The 12-month PFS was 20.8% (95% CI: 5.2–43.6%) for del17p, 27.8% (95% CI: 11.7–46.6%) for OHRC, and 15% (95% CI: 3.7–33.5%) for standard-risk cytogenetics.

In patients who received Selinexor-based bridging therapy, the median PFS was 13.1 months (95% CI: 0.7-Not Reached) with 6-month PFS of 70% (95% CI: 32.9–89.2%) and 12-month PFS of 58.3% (95% CI: 23–82.1%). Whereas in patients who did not receive Selinexor-based bridging therapy, the median PFS was 2.1 months (95% CI: 1.4–4.6) with 6-month PFS of 26.7% (95% CI: 15.2–39.7%) and 12 months PFS of 13.4% (95% CI: 5.5–24.7%).

Overall survival was similar among the cohorts (*p* = 0.920). In patients with del17p, the median overall survival (OS, Figure 2B) was 10.9 months (95% CI: 3.4–25.5) with 6-month survival of 68.2% (95% CI: 39.5–85.4%) and 12-month survival of 40.9% (95% CI: 17.1–63.6%). Similar trends were noted in patients with OHRC and standard-risk cytogenetics with a median survival of 10.3 months in both groups. The 6-month survival was 70.8% (95% CI: 48.4–84.9%) and 12-month survival was 42.9% (95% CI: 22.4–61.9%) in patients with OHRC. In patients with standard-risk cytogenetics, the 6-month survival was 70% (95% CI: 45.1–85.3%) and 12-month survival was 45% (95% CI: 23.1–64.7%).

In the overall study cohort, the patients who received a Selinexor-based regimen as a bridge to an advanced therapy (typically a CAR-T) had a numerically improved median overall survival (15.5 months; 95% CI: 7.4-not reached) with a 6-month survival of 100% and 12-month survival of 67.8% (95% CI: 29.1–88.2%). In patients who received Selinexor for other indications rather than as a bridge, the median survival was 9 months (95% CI: 5.7–13.7), with 6-month survival of 63.8% (95% CI: 48.9–75.5%) and 12-month survival of 38.3% (95% CI: 24.8–51.7%).

## 4. Discussion

This analysis includes 60 patients with RRMM who were treated with a Selinexor-based regimen. To our knowledge, this is the first reported retrospective analysis specifically examining the use of Selinexor in TP53-altered RRMM patients. Our study’s primary purpose was to investigate the efficacy of Selinexor in RRMM pts with del17p as compared to pts with OHRC and standard-risk cytogenetics. The deletion of chromosome 17p, which contains the TP53 gene, is associated with a poor outcome in terms of PFS and OS in pts with RRMM [24]. A pooled analysis of 1064 patients enrolled on IFM trials, which included 11% with del17p, showed that this abnormality negatively impacted both the event-free and overall survivals. The median event-free survival for del17p pts was 15 months versus 35 months for patients without any genomic aberration (*p* < 0.001) [24]. Conversely, in our study the objective response was not statistically worse in patients with del17p, and the trend suggested an improved ORR with a Selinexor-based regimen (50% vs. 41.7% vs. 35% in pts with del17p, OHRC and standard-risk cytogenetics, respectively). Depth of response were similar across all the groups. In a post hoc analysis, median PFS was numerically higher for the del17p group vs. the other groups (4.1 months vs. 1.5 months vs. 2.6 months in pts with del17p, OHRC, and standard-risk cytogenetics, respectively); however, our patient population did include a significant portion who used Selinexor only as a bridging therapy, so further studies are needed to confirm this finding. Our study also suggests that Selinexor is an effective bridging therapy to other advanced therapies (typically CAR-Ts in our dataset) with a median OS of 15.5 months for patients who received Selinexor as a bridging therapy versus 9 months for Selinexor use for other reasons rather than as a bridge. The 6- and 12-month overall survival rates were also higher in patients who received a Selinexor-based bridging therapy. These data suggest that a Selinexor-based regimen is a reasonable bridging therapy for high-risk, heavily pretreated patients waiting for subsequent line of therapies, especially T-cell redirecting therapies. Selinexor has also previously been shown to have minimal effect in collecting and stimulating the T-cells for cellular therapy purposes, further supporting this indication [25].

Our results did not show any significant PFS and OS differences in del17p pts treated with Seli-based regimens, but it did suggest improved outcomes in some comparison and compared to historical controls. This effect is more pronounced given the fact that most of the patients (13 out of 16) with del17p had an additional high-risk cytogenetic abnormality (three patients had t(4;14) and ten patients had a gain or amplification of 1q21). In a prior study of RRMM patients treated with Selinexor as a salvage therapy after progression on a BCMA-directed CAR-T therapy, the ORR was 40% as first-line salvage therapy and 21.4% at any line of salvage therapy with the use of Selinexor-based regimens [26]. Similarly, in the Phase II multicenter prospective STORM (Selinexor Treatment of Refractory Myeloma) trial, 79 RRMM patients were treated with Selinexor in combination with dexamethasone. There were eight patients with del17p alone and four patients with del17p along with high-risk cytogenetics. The ORR was 38% for del17p patients treated with Selinexor plus dexamethasone as compared to 35% for all of the high-risk patients, 21% for entire cohort, and 18% for patients with standard-risk cytogenetics [27]. The phase 2b portion of the STORM trial evaluated the efficacy of Selinexor 80 mg twice weekly with Dexamethasone in 122 penta-refractory multiple myeloma patients. There were 65 patients (53%) with high-risk cytogenetics, including del17p 32 (26%), t(4:14) 17 (14%), t(14:16) 5 (4%), and gain (1q) 40 (33%) patients. A minimal response or better was observed in 39% of patients for the entire cohort as compared to patients with high-risk cytogenetics, where a minimal response or better was noted in 37% of patients. The ORR for the entire population was 26% with a median PFS of 3.7 months (95% CI, 3.0 to 5.3) and median OS of 8.6 months (95% CI, 6.2 to 11.3). Whereas in patients who had a partial response or better, or a minimal response or better, the median overall survival was 15.6 months [22]. Similarly, in the Phase III BOSTON trial (Bortezomib, Selinexor and Dexamethasone in patients with Multiple Myeloma), which randomized patients 1:1 to Bortezomib and Dexamethasone ± Selinexor (at 100 mg/week). There were 195 patients who received the Selinexor-based regimen. Half (n = 97) of the patients in the Selinexor arm had high-risk cytogenetics: 11% (n = 21) with del17p, 41% (n = 80) with 1q21 amplification, 4% (n = 7) with t(14:16), and 11% (n = 22) with t(4:14). The ORR was 76% for the entire cohort treated with a Selinexor-based regimen versus 62% in patients treated with Bortezomib/Dexamethasone. In the high-risk cytogenetic patients treated with a Selinexor based regimen, the ORR was higher at (77.3% [67.7–85.2] vs. 55.8% [45.2–66.0]; 2.70 [1.4–5.0], *p* = 0.0008), compared to patients treated with Bortezomib/Dexamethasone. The benefit was particularly strong for patients with del17p with a hazard ration of 0.38 (95% Cl, 0.16–0.86)

Our findings and earlier prior studies with Selinexor-based regimens support the use of Selinexor-based therapies in the patients with high-risk cytogenetics, particularly with del17p and particularly as a short-term bridging therapy, given its initial PFS benefit in this population. We hypothesize that this improvement is due to Selinexor’s novel mechanism of action which should have additional benefit in these patients.

The main limitation of our study is its retrospective design. A second limitation is the relatively small number of patients included in all groups. Finally, the population is highly heterogeneous and were treated with various Selinexor combinations which could certainly lead to some confounding of our data.

## 5. Conclusions

Overall, Selinexor-based regimens showed promising responses even in a heavily pretreated, high-risk population. Our analysis, while only representing a small patient population, suggests that Selinexor-based regimens lead to at least similar outcomes among RRMM patients with del17p compared to patients with OHRC. This contrasts with several studies of combinations of novel therapies in this population, where the del17p patients often have a poorer prognosis. Interestingly, our data also suggest that using a Selinexor-based regimen appears to have some initial PFS benefit in del17p patients for 4–6 months, which suggests its utility as a bridging regimen in patients waiting for advanced therapies, particularly CAR-T cells. Further investigation into this population is warranted, including in earlier lines of therapy, in hopes of seeing a more durable response.

## Figures and Tables

**Table 1:** Baseline characteristics of included population.

	del17p (N = 16)		OHRC (N = 24)		Standard Risk (N = 20)	
	N	%	N	%	N	%
**Gender**						
Male	10	62.5	15	62.5	13	65.0
Female	6	37.5	9	37.5	7	35.0
**Race**						
White	13	81.3	14	58.3	16	80.0
Black	3	18.8	10	41.7	4	20.0
**Median Age in Years (Range)**	62.5	46–81	69	44–83	65.5	51–90
**High Risk Features**						
del17p	16	100	0	0.0	0	0.0
t(4;14)	3	18.8	5	20.8	0	0.0
t(14;16)	0	0.0	3	12.5	0	0.0
t(14;20)	0	0.0	1	4.2	0	0.0
Gain or Amp 1q21	10	62.5	22	91.7	0	0.0
**ISS at diagnosis**						
I	1	6.3	2	8.3	4	20
II	6	37.5	10	41.7	8	40
III	6	37.5	11	45.8	2	10
Unknown	3	18.6	1	4.2	6	30
**R-ISS at diagnosis**						
I	1	6.3	1	4.2	3	15
II	7	43.8	12	50.0	9	45
III	5	31.3	7	29.2	0	0
Unknown	3	18.8	4	16.7	8	40
**Median No. of prior lines of therapy (range)**	4	3–11	5	3–12	5	3–16
**Prior Transplant**	11	68.8	15	62.5	14	70.0
**Prior Lenalidomide**	15	93.8	22	91.7	20	100.0
Refractory to Lenalidomide	15	100.0	18	81.8	19	95.0
**Prior Bortezomib**	16	100.0	24	100.0	20	100.0
Refractory to Bortezomib	16	100.0	21	87.5	15	75.0
**Prior Carfilzomib**	15	93.8	21	87.5	18	90.0
Refractory to Carfilzomib	14	93.3	19	90.5	16	88.9
**Prior Pomalidomide**	16	100.0	21	87.5	19	95.0
Refractory to Pomalidomide	16	100.0	20	95.2	19	100.0
**Prior anti-CD38 antibody**	16	100.0	24	100.0	19	95.0
Refractory to anti-CD38 antibody	16	100.0	23	95.8	18	94.7
**Time since MM diagnosis, years**						
Median (Range)	5.6	0.7–25.2	4.1	0.8–11.5	4.8	1.0–11.2
**Follow up, months**						
Median (Range)	10.5	2.5–33.0	8.4	0.6–49.1	10.3	0.7–54.5

**Table 2:** Selinexor-containing regimens used in included population.

	del17p (N = 16)		OHRC (N = 24)		Standard Risk (N = 20)	
	N	%	N	%	N	%
**Dara-Seli**	0	0.0	0	0.0	1	5.0
**Dara-Seli-dex**	2	12.5	6	25.0	2	10.0
**Seli-Kd**	5	31.3	7	29.2	10	50.0
**Seli-dex**	1	6.3	5	20.8	2	10.0
**Seli-pom**	0	0.0	1	4.2	0	0.0
**Seli-Pd**	5	31.3	2	8.3	3	15.0
**Seli-Vd**	3	18.8	3	12.5	2	10.0

Dara—Daratumumab, Seli—Selinexor, Dex—Dexamethasone, d—Dexamethasone, K—Carfilzomib, pom—Pomalidomide.

## Data Availability

Data available per request to corresponding author.
